# Therapeutic potential of human stem cell transplantations for Vanishing White Matter: A quest for the Goldilocks graft

**DOI:** 10.1111/cns.13872

**Published:** 2022-07-01

**Authors:** Anne E. J. Hillen, Prisca S. Leferink, Nicole B. Breeuwsma, Stephanie Dooves, Talia Bergaglio, Marjo S. Van der Knaap, Vivi M. Heine

**Affiliations:** ^1^ Department of Pediatrics and Child Neurology, Amsterdam Neuroscience, Emma Children's Hospital Amsterdam UMC Location Vrije Universiteit Amsterdam Amsterdam the Netherlands; ^2^ Department of Child and Adolescence Psychiatry, Amsterdam Neuroscience, Emma Children's Hospital Amsterdam UMC Location Vrije Universiteit Amsterdam Amsterdam The Netherlands; ^3^ Center for Neurogenomics and Cognitive Research, Vrije Universiteit Amsterdam, Amsterdam Neuroscience, Department of Complex Trait Genetics Amsterdam UMC Location Vrije Universiteit Amsterdam Amsterdam The Netherlands; ^4^ Department of Functional Genomics, Center for Neurogenomics and Cognitive Research, Vrije Universiteit Amsterdam, Amsterdam Neuroscience Amsterdam UMC Location Vrije Universiteit Amsterdam Amsterdam The Netherlands

**Keywords:** cell transplantation, glia, human pluripotent stem cells, leukodystrophy, Vanishing white matter

## Abstract

**Introduction:**

Vanishing white matter (VWM) is a leukodystrophy that leads to neurological dysfunction and early death. Astrocytes are indicated as therapeutic target, because of their central role in VWM pathology. Previous cell replacement therapy using primary mouse glial precursors phenotypically improved VWM mice.

**Aims:**

The aim of this study was to determine the translational potential of human stem cell‐derived glial cell replacement therapy for VWM. We generated various glial cell types from human pluripotent stem cells in order to identify a human cell population that successfully ameliorates disease hallmarks of a VWM mouse model. The effects of cell grafts on motor skills and VWM brain pathology were assessed.

**Results:**

Transplantation of human glial precursor populations improved the VWM phenotype. The intrinsic properties of these cells were partially reflected by cell fate post‐transplantation, but were also affected by the host microenvironment. Strikingly, the spread of transplanted cells into the white matter versus the gray matter was different when grafted into the VWM brain as compared to a healthy brain.

**Conclusions:**

Transplantation of human glial cell populations can have therapeutic effects for VWM. For further translation to the clinic, the microenvironment in the VWM patient brain should be considered as an important moderator of cell replacement therapy.

## Graphical abstract

Intracranial transplantation of human stem cell‐derived glial progenitor populations improves the leukodystrophy phenotype of Vanishing white matter (VWM) mice. In the VWM brain, transplanted cells show altered migration and cell fate as compared to cells transplanted into a healthy brain. Translation to the clinic should consider the microenvironment in the VWM brain as an important moderator of cell replacement therapy.

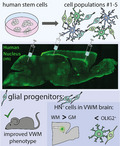

## 1 INTRODUCTION

Vanishing white matter (VWM) is a leukodystrophy caused by recessive mutations in the genes encoding the subunits of eukaryotic translation initiation factor 2B (eIF2B).^1^ VWM causes motor deficits, cognitive decline, and epilepsy. The age of onset is a determinant of disease severity, with the most severe phenotypes having a prenatal or infantile onset.^2^, ^3^, ^4^ Episodes of rapid and major decline are provoked by febrile infections, minor head trauma, or acute fright. Such episodes may lead to coma and early death, or are followed by partial recovery.^3^ No curative treatments are presently available.

Neuropathology studies show that VWM leads to vacuolization and cystic degeneration of the white matter, with little microglial or neuronal involvement or deterioration.^5^, ^6^, ^7^ Astrocytes in VWM patients and mice are dysmorphic, immature, display perturbed reactivity,^5^ and are suggested to be the primary affected cell type in the brain.^6^, ^8^ Why astrocytes show increased susceptibility to mutations in the subunits of eIF2B is not fully understood. eIF2B plays a role in translation initiation and can be downregulated in response to various stress conditions that activate the integrated stress response (ISR).^9^ Recent studies confirm a constitutively increased ISR activation in especially astrocytes of the VWM brain.^8^, ^10^ The identification of astrocytes as the likely culprit of VWM pathology marks this cell population as a target for potential therapies.

Transplants of healthy mouse glial progenitor cell populations into VWM mutant mice showed promising results.^11^ The success rate was determined by the degree to which transplanted cells became GFAP^+^ astrocyte lineage cells,^11^ providing prospects for astrocyte replacement therapies in human patients. With the introduction of induced pluripotent stem cell (iPSC) technology,^12^, ^13^ we are now able to generate autologous cell transplants for regenerative medicine purposes. Indeed, stem cell replacement therapies have used neural precursor cells (NPCs)^14^ and dopaminergic neurons^15^, ^16^ in Parkinson's disease, NPCs in neurospheres for spinal cord injury,^17^ melanocytes in a pigmentation model for Vitiligo,^18^ and retinal tissue differentiations.^19^ Here, we transplanted various human pluripotent stem cell‐derived glial cell populations^20^ into immunocompromised VWM mice, in order to test the translational potential of these populations in a cell replacement therapy for VWM.

## 2 MATERIALS AND METHODS

### 2.1 Animals

*Eif2b5* ^R191H^ *Rag2* ^KO^ mice with a C57BL/6J background were used as an immunocompromised VWM mouse model. *Eif2b5* homozygous mice develop VWM, whereas heterozygous littermates do not and were used as healthy controls.^6^ All pups of one litter received the same transplantation condition, considering each animal an experimental unit. Litters were randomly assigned to a transplantation condition. All animals were housed in the same location. The animal ethical committee of Amsterdam UMC, the Netherlands, approved all animal experiments. ARRIVE 2.0 guidelines^21^ were considered for the reporting on animal‐related data in this manuscript.

### 2.2 Generation of cell populations

Human ESCs (hESC line H01) were differentiated into glial (precursor) cells as described in Nadadhur et al.,^20^ using a retinoic acid (RA)‐based glial progenitor induction. For detailed methods, see Appendix S1. Briefly, cells were cultured until day 53 in differentiation medium to obtain glial precursor cells (population #1, or Pop1). We additionally tested 4 other cell populations (Table S1). These populations included an iPSC‐derived matured astrocyte population (#2); iPSC‐derived astrocytes (#3); and hESC‐derived astrocytes induced with FGF2 (#4). Lastly, the culturing procedure of Pop1 cells was repeated using various iPSC lines, and matured until days 60–105 (median 77) to generate a glial population with a more mature phenotype (population #5, or Pop5).

### 2.3 Injection of cells into neonatal mice pups

*Eif2b5* ^R191H^ *Rag2* ^null^ mouse neonates were injected in the brain as previously described.^11^ In brief, cells were detached from the plate using Accutase (Innovative Cell Technologies, Inc., San Diego, CA) and spun down. Cells were resuspended in 0.4 μl saline with 100 μg/ml DNAse‐I to a concentration of 250,000 cells/μl and stored on ice until use. P0 pups were anesthetized by hypothermia and injected using a Hamilton syringe and pump. 100,000 cells in 400 nl of cell suspension were injected per injection site, at a flow rate of 1000 nl/min, bilaterally in the genu and splenium of the corpus callosum and in the cerebellum. For control groups, animals were injected with 400 nl of saline per injection site. After injection, pups were placed on a heat mat and allowed to recover before returning to the nest.

### 2.4 Assessment of VWM pathology in adult mice

Animals were weighed from 4 months of age until culling. The researchers were blinded to the treatment conditions during all assessments of animals for VWM pathology as described previously in Dooves et al.^6^ Briefly, at 7 months of age motor tests were performed. Mice were tested on grip strength and accuracy and latency of crossing a balance beam. To provide sufficient rest for the animals between motor experiments, litters were tested in fixed order while the order of individual mice per cage was at random. Brain tissue was collected at 2, 5, and 8–9 months of age. Tissue collected at 8–9 months was used to investigate pathology in the brain as described previously, that is, translocated Bergmann glia in the cerebellum, Nestin^+^GFAP^+^ cells in the corpus callosum, and *Plp* ^+^ cells in the white matter of the corpus callosum and cerebellum. While cognitive signs have been noted in VWM patients, especially in late‐onset cases,^3^ the VWM mouse model does not show striking cognitive deviations from healthy controls.^6^ Therefore, effects of cell replacement therapy on cognitive recovery have not been assessed in this study. Similarly, although cell replacement therapy has been found to decrease neuroinflammation,^22^, ^23^ this was not investigated in the current study; neuroinflammation in the VWM brain is lacking in relation to the structural white matter damage, with meager astrogliosis,^5^ axonal damage secondary to astrocytic pathology,^24^ and no significant microglial activation.^5^, ^6^

### 2.5 Pathology quantification

RT‐PCR, immunocytochemistry, immunohistochemistry, and in situ hybridization procedures were used to determine VWM brain pathology (Tables S2–S4). A Leica DM5000B was used to visualize IHC and ISH data. Images were taken at 100× magnification. To determine VWM pathology in the Bergmann glia of the cerebellum, in Nestin^+^ astrocytes in the corpus callosum, and in *Plp* ^+^ cells in the brain white matter, cell counts were performed as described previously.^6^, ^11^ When appropriate, cells counterstained with dapi were counted in an automated fashion using ImageJ (ICTN plug‐in, settings 14, 5, 0.1). Researchers were blinded to the treatment condition of the analyzed animals during all counts.

An image was taken of the genu portion of the corpus callosum, of a portion of cortex adjacent dorsally, and of the most superficial area of the cortex dorsal to that. The splenium portion of the corpus callosum was also imaged. For each animal, 3 to 4 brain sections were imaged. The sections analyzed were as similar as possible between animals with regard to medial‐lateral location. The numbers of HN^+^ cells counted in these images were used to calculate the ratio of HN^+^ cells in the white matter and the gray matter. No HN^+^ WM/GM ratios were calculated for two animals in the Pop5 group (1 control and 1 VWM) that had received a mislocalized injection, as this confounded cell localization results. No other data points of individual assays were excluded. To assess the cell fate of transplanted cells, the numbers of OLIG2^+^HN^+^ or SOX9^+^HN^+^ were divided by the total number of HN^+^ cells in the genu and splenium.

### 2.6 Statistics

Data were analyzed with SPSS software (IBM SPSS Statistics 26.0). A binary logistic regression model was used to study the overall phenotype of VWM mutant animals that received cell transplantations (Table S5). The model was based on data of (homozygous) VWM and (heterozygous) healthy control animals injected with saline. The regression analysis can only be run on animals with no missing data on the predictor variables; 16 of 19 saline‐treated controls and 15 of 16 saline‐treated VWM animals were included. These groups differed significantly from one another on each variable included in the model. Variables included pathology assays (Bergmann glia translocation, Nestin^+^GFAP^+^ astrocytes in the corpus callosum, and *Plp* ^+^ cells in the cerebellar white matter and the corpus callosum) and motor skill assays (grip strength and accuracy and speed of traversing a balace beam). The variance inflation factor (VIF) of each predictor variable was below 5, indicative of no multicollinearity among variables. To ensure this assumption was met, the variance proportions were also inspected, which showed no values of >0.9 were present.

All data were tested for normality using Shapiro–Wilks tests. To compare groups, independent t‐tests or one‐way ANOVAs with Tukey's post hoc tests were performed when appropriate. A two‐way repeated‐measures ANOVA was used to compare *Eif2b5* ^R191H^ genotypes on within‐subject variables when appropriate. Welch tests with Games–Howell post hoc tests were used in case of unequal variances as indicated by a Levene's test. Non‐parametric alternatives used were Mann–Whitney *U*‐test or Kruskal–Wallis tests.

## 3 RESULTS

A proof‐of‐principle study on cell replacement therapy for VWM showed success by transplanting primary mouse glia progenitor cells into a VWM mouse model.^11^ This study showed that the degree of transplanted cells that adopted an astrocytic cell fate in vivo was significantly higher in improved animals than in non‐improved animals. To study the translational potential of this therapeutic approach, we transplanted various human glial populations and analyzed their regenerative potential.

### 3.1 Migration and cell survival of transplanted human glial cell populations in VWM white matter

**FIGURE 1:**
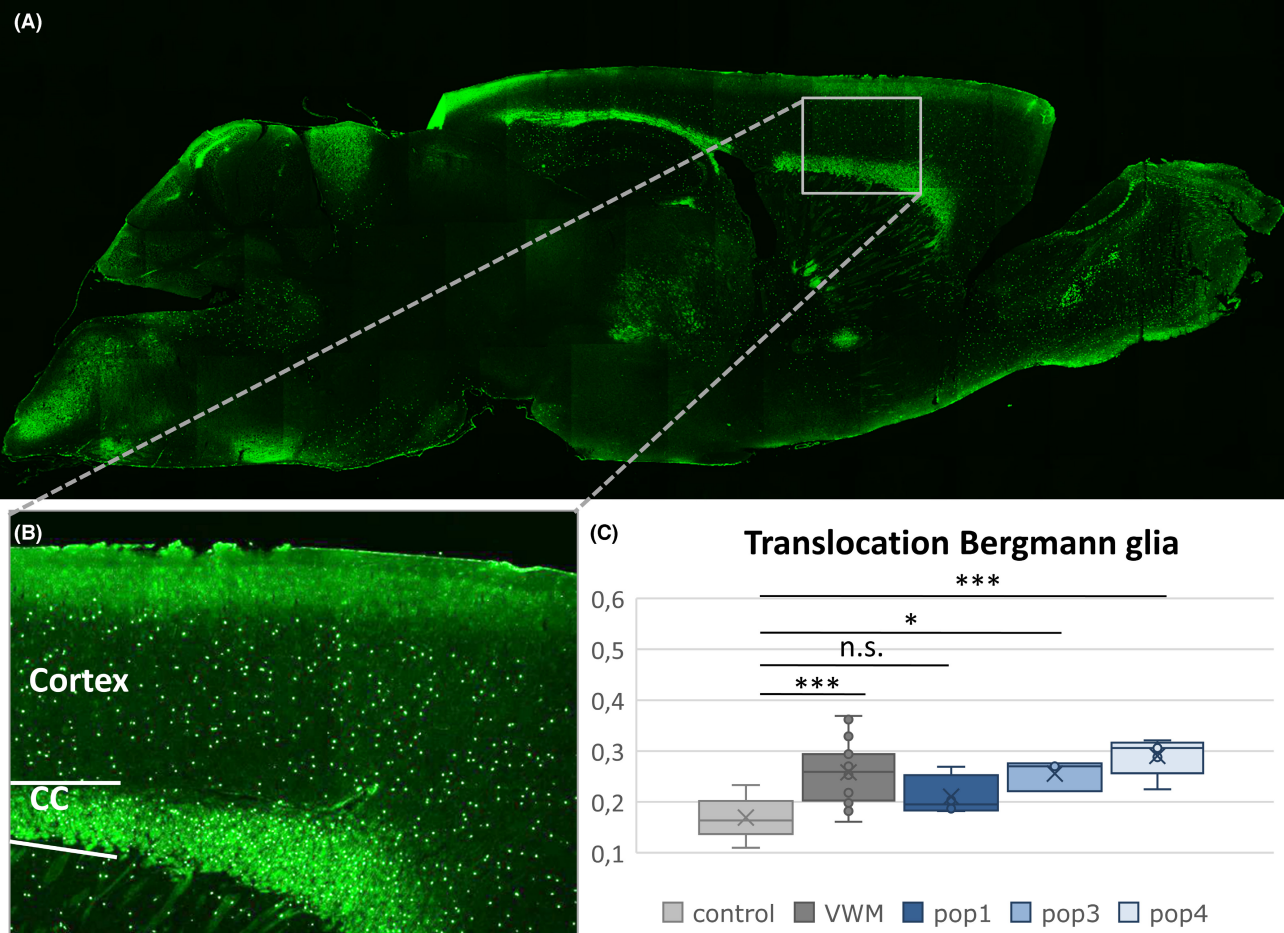
Spread of transplanted cells and their effect on Bergmann glia translocation. Example of migration of cells in a VWM animal at 5 months of age (A) with a close‐up of the cortex and corpus callosum (B). One‐way ANOVA analysis of VWM groups (saline‐treated (*n* = 26), Pop1 (*n* = 4), Pop3 (*n* = 3), Pop4 (*n* = 5)) shows that Bergmann glia translocation at 9 months of age was significantly increased as compared to saline‐treated healthy controls (*n* = 28), except for Pop1‐treated mutants (C). n.s.: non‐significant; *p < 0.05; ***p < 0.001

We selected culturing protocols that differentiate human pluripotent stem cells into various glial cell populations, ranging from a precursor population to mature astrocytes (Table S1). To determine which human glial populations are suitable for transplantation, cells were bilaterally injected at birth into the white matter of an immunosuppressed VWM mouse model. Transplantations included the following glial precursor cells: glial precursors generated via retinoic acid (RA)‐induction (Population #1 or Pop1); mature astrocytes (Pop2); and intermediate glial cell precursors (Pop3 and Pop4, see Figure S1). To identify the xenograft cells in vivo, we performed immunohistochemistry for the Human Nucleus (HN) antigen. At five months post‐transplantation, HN^+^ cells had migrated throughout the brain, including to the olfactory bulb, the corpus callosum, cortex, hypothalamus, hippocampus, midbrain, cerebellum, and brainstem (Figure 1A,B). This was observed for all populations, except for Pop2. These cells showed a mature astrocyte expression profile upon injection but could not be traced back in the brain at 5 months of age, indicating poor cell survival in vivo (data not shown). For this reason, these animals were excluded from further analysis. In contrast, we successfully transplanted the remaining four human glia populations into a VWM mouse model and showed widespread migration and cell survival of transplanted populations.

### 3.2 Evaluation of the effect of cell transplantations on the VWM phenotype

To study the effects of cell transplantations on disease severity in animals in adulthood, we inspected the number of translocated Bergmann glia (BG) in the cerebellum. BG translocation is a disease marker of VWM and is sensitive to therapeutic intervention.^25^ We found that of the culturing protocols tested, only animals transplanted with Pop1 cells showed an improvement in BG translocation toward healthy control levels (*F* = 14.01, *p* < 0.001, Pop1 vs. control post hoc non‐significant) whereas Pop3 (vs. control post hoc *p* = 0.040) and Pop4 (vs. control post hoc *p* < 0.001) performed comparably to VWM saline‐treated animals (vs. control post hoc *p* < 0.001; Figure 1C). We therefore used the Pop1 protocol to generate another population of these cells, referred to as Pop5, to transplant into a larger group of animals.

**FIGURE 2:**
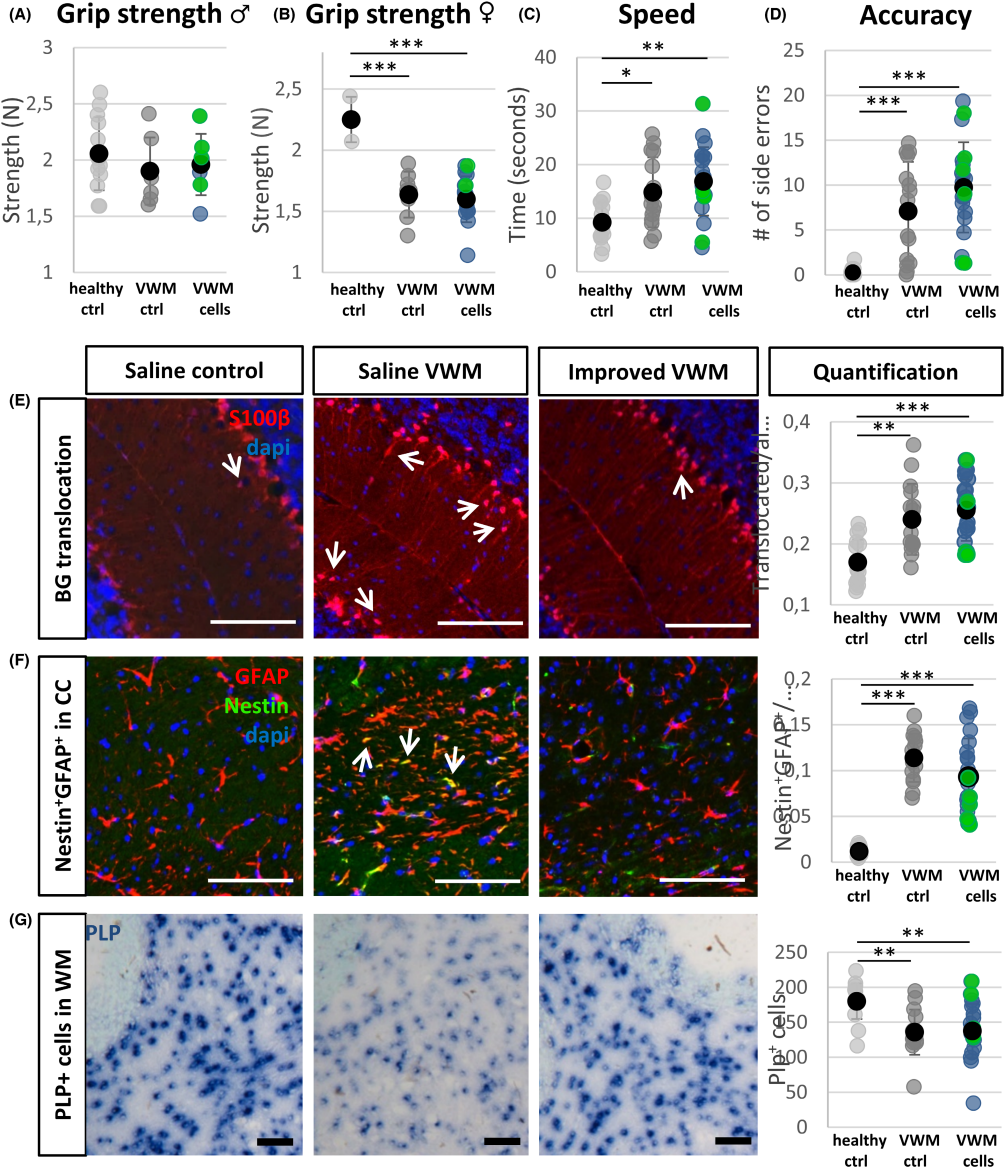
Outcomes of cell‐treated animals on the variables included in the regression model. The black dot with error bars represents the group mean ± standard deviation. Green dots indicate animals that were classified by the regression model as improved. Graphs depict performance of 7‐month‐old animals on the grip strength in the front and hind paws of male (A) and female (B) animals, and speed (C) and accuracy (D) of crossing a narrow beam. Representative images and quantification of improvement in the number of translocated Bergmann glia in the cerebellum (arrows, E), Nestin^+^GFAP^+^ cells in the corpus callosum (arrows, F), and *Plp* ^+^ cells in the cerebellum (G; graph also includes PLP^+^ cells in the corpus callosum) of animals 9 months of age. The Cells VWM group (*n* = 21) includes animals transplanted with pop1 (*n* = 4), pop3 (*n* = 5), pop4 (*n* = 3), and pop5 (*n* = 9), and was compared with healthy controls (*n* = 16) or VWM mice (*n* = 15) treated with saline. Groups were compared in one‐way ANOVAs with Tukey's post hoc tests in B–D and F–H. For E, a Kruskal–Wallis test was used. *p < 0.05; **p < 0.01; ***p < 0.001. Scalebars: 100 μm

To judge in more detail whether the VWM phenotype had improved after transplantation with Pop1 or Pop5 cells, various phenotypic assays were used as validated by Dooves et al.^6^ (Figure 2). These include tests of motor skills such as grip strength (females *F*[2,49] = 8.717, *p* = 0.001; males non‐significant; Figure 2A,B), balance beam speed (*F*[2,49] = 8.232, *p* = 0.001; Figure 2C) and accuracy (*H* (2) = 29.087, *p* < 0.001; Figure 2D), and VWM pathology markers, including BG translocation in the cerebellum (*F*[2,49] = 14.753, *p* < 0.001; Figure 2E), Nestin^+^ astrocytes in the corpus callosum (*F*[2.23,404] = 140.207, *p* < 0.001; Figure 2F) and *Plp* ^+^ cells in the brain white matter (*F*[2,49] = 9.325, *p* < 0.001; Figure 2G; Figure S2). As group analysis can obscure improvements of individual mice,^11^, ^26^ these measurements of pathology and motor skills were used as predictor variables in a logistic regression model. This model calculates the probability of the performance of a transplanted animal being classified as (resembling) “(saline‐treated) VWM” or “(saline‐treated) healthy control” (also see Table S5). In the case of missing data, animals were excluded from the regression analysis. The regression was based (trained) on data of the saline‐injected animals and therefore correctly classified the genotypes of all animals in this treatment group. The logistic regression model explained 75% (Cox & Snell *R* ^2^ = 0.750) of the variance in pathology outcome, which was statistically significant (Chi‐squared *χ* ^2^ (6) = 44.361, *p* < 0.001). A Hosmer & Lemeshor test was not significant, indicating good fit of the model. The model was used to determine a classification of the performance of cell‐injected VWM animals as either “saline‐injected VWM” or “saline‐injected healthy control.” Healthy controls injected with cells were excluded from analysis. A cut‐off score of <0.25 was used for the probability of being assigned as a saline‐injected VWM animal. The model classified 46.1% (6 out of 13) of VWM animals transplanted with Pop1 or Pop5 cells as performing similarly as healthy controls, indicating human glial transplants can improve the phenotype in a VWM mouse model. Table 1 presents an overview of the performance of these improved animals on various VWM assays, showing that improved animals transplanted with Pop1 or Pop5 outperformed the group average of saline‐treated VWM animals on at least 3 VWM disease parameters.

**TABLE 1:** Overview of regression probability and performance on each predictor variable by mutant mice that improved after transplantation

	Classification by regression model	Probability in regression model	Nestin^+^ astrocytes in corpus callosum	Translocated Bergmann glia	Plp^+^ cells	Grip strength	Balance beam accuracy	Balance beam speed
*Control saline M (n = 19)*	WT	1.00000	0.012 ± 0.01	0.168 ± 0.04	180.9 ± 24.91	2.1 ± 0.31	0.27 ± 0.45	8.8 ± 3.67
**VWM saline M (*n* = 16)**	VWM	1.00000	0.113 ± 0.26	0.239 ± 0.56	137.7 ± 32.16	1.8 ± 0.26	7.04 ± 5.32	14.8 ± 6.31
Pop1 M (*n* = 4)	–		0.087 ± 0.03	0.210 ± 0.04	154.0 ± 26.28	1.9 ± 0.54	10.9 ± 6.99	11.4 ± 4.03
Pop5 M (*n* = 9)	–		0.730 ± 0.04	0.255 ± 0.05	133.3 ± 48.75	1.7 ± 0.18	9.3 ± 3.83	17.1 ± 6.16
A (pop1)	WT	0.21889	**0.092**	**0.187**	**140**	**2.02**	18	**14**
B (pop1)	WT	0.00787	**0.063**	0.269	130	***2.11***	13	**14**
C (pop1)	WT	0.00002	**0.071**	**0.182**	***190***	***2.39***	**1.33**	**5.5**
D (pop5)	WT	0.18313	**0.042**	0.337	106.4	**2.08**	**1.78**	**11.7**
E (pop5)	WT	0.10567	**0.041**	**0.183**	127.6	**1.87**	9.0	16.7
F (pop5)	WT	0.00310	**0.047**	**0.182**	**141.5**	1.71	11.0	15.0

*Note*: Bold: better than VWM saline average. Italic: better than WT saline average.

### 3.3 In vitro characterization of cell populations that improve the VWM phenotype

**FIGURE 3:**
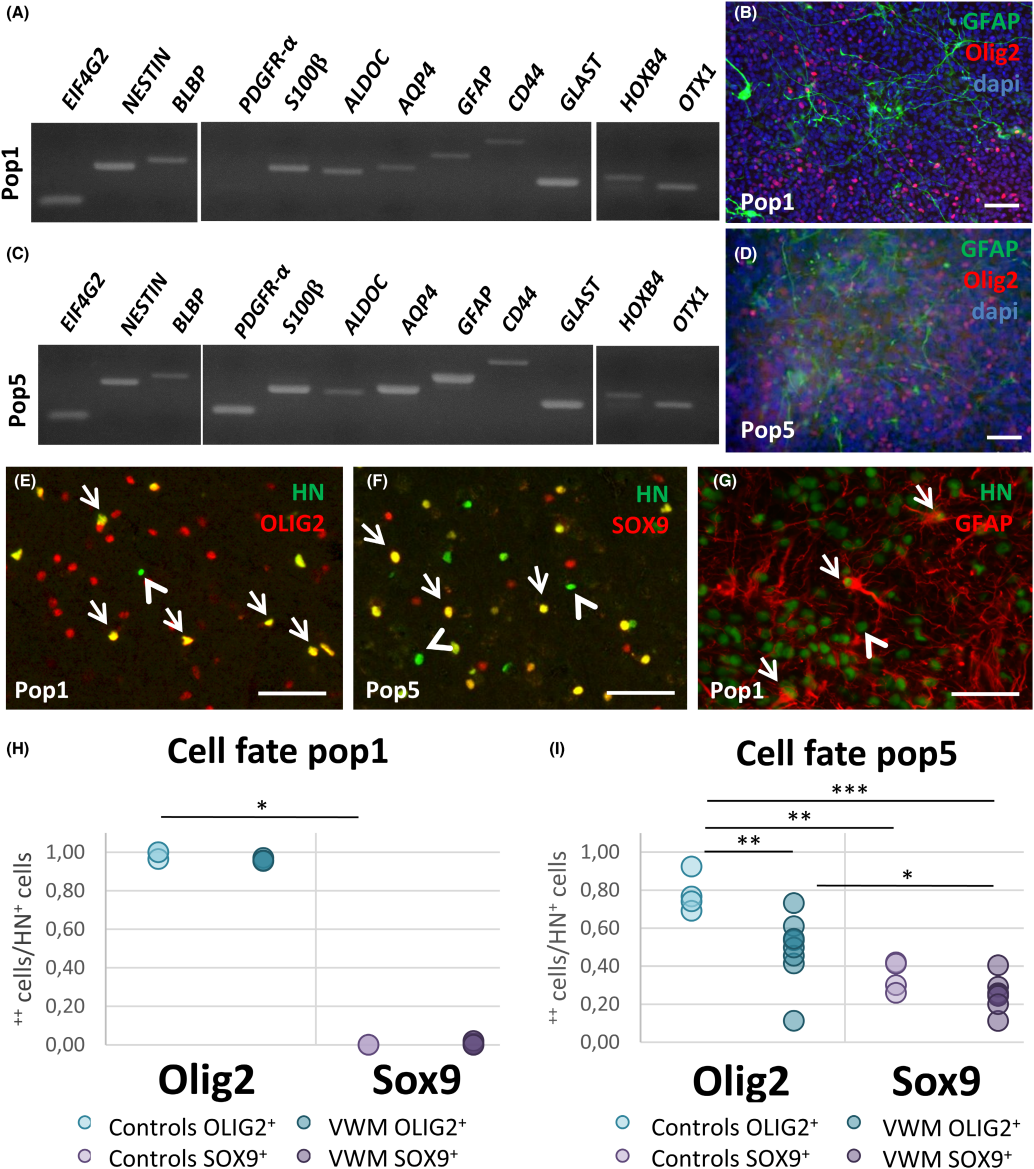
In vitro and in vivo characteristics of cell populations with therapeutic potential. In vitro characterization was performed by RT‐PCR (A, C) and immunohistochemistry (B, D) of the Pop1 cell population (A, B) and Pop5 cell population (C, D). Scale bars represent 50 μm. Transplanted HN^+^ cells co‐localized with OLIG2 (E), SOX9 (F), and GFAP (G) in vivo at 8–9 months of age. The ratio of OLIG2^+^HN^+^ or SOX9^+^HN^+^ cells over the total number HN^+^ cells in the white matter was determined for 8‐month‐old healthy controls (*n* = 3) or VWM animals (*n* = 4) receiving Pop1 cells (H), and for 9‐month‐old healthy controls (*n* = 4) or VWM animals (*n* = 7 for OLIG2, *n* = 5 for SOX9) receiving Pop5 cells (I). Kruskal‐Wallis test was used in H. A one‐way ANOVA was used in I. *p < 0.05; **p < 0.01; ***p < 0.001. Scale bars: 100 μm

The glial cells that improved disease severity upon transplantation were derived using a culturing protocol based on a RA‐based induction. At the differentiation stage, Pop5 cells were maintained longer in culture than Pop1 cells, possibly affecting their fate. By contrast, Pop2 cells were switched to different media, that is, commercial astrocyte medium, to establish a more astrocytic culture. However, Pop2 cells did not appear to survive post‐transplantation. To characterize the transplanted human glial populations, we performed an RT‐PCR and immunocytochemistry on the cells prior to transplantation (also see Figure S1). Both Pop1 and Pop5 revealed expression of various glial progenitor, astroglial, and oligodendrocytic genes, and confirm a mix of astrocytes and oligodendrocyte lineage cells (Figure 3A–D). Pop1 cells showed a high expression of *NESTIN*, *BLBP*, *GLAST*, and OLIG2, in combination with the less strongly expressed *GFAP* and *AQP4* genes, indicating that the astrocytes (progenitors) in this culture are relatively immature (Figure 3A,B). Pop5 cells showed similar gene expression in vitro as Pop1 cells, but with higher expression levels of mature astrocyte markers such as *GFAP* and *AQP4*, in line with in vitro procedures. The oligodendrocyte lineage fate was indicated by *PDGFaR* expression by RT‐PCR (Figure 3C). The presence of oligodendrocyte precursors was further confirmed by OLIG2 expression (Figure 3D). Thus, while both populations contained a mix of astrocytic, oligodendrocytic, and glial progenitor cells, Pop5 cells showed higher expression of astrocytic lineage‐related genes, possibly induced by prolonged maintenance in vitro. Taken together, we selected an in vitro procedure that successfully generated a cell population that proved fit for VWM cell replacement therapy in close to half of transplanted animals.

### 3.4 Cell fate of transplanted cells in vivo

In order to investigate the gliogenic properties of the cells post‐transplantation, HN^+^ cells were analyzed for co‐localization either with OLIG2 to indicate an oligodendrocytic lineage (Figure 3E) or with SOX9 or GFAP to indicate an astrocytic lineage (Figure 3F,G). At 2 months after injection, cells remained clustered at the injection sites and <10% of HN^+^ Pop1 cells were OLIG2^+^ (Figure S3A,C,F). A sharp increase in cellular spread and proportion of OLIG2^+^HN^+^ cells was seen at 5 months, when nearly all HN^+^ cells had become OLIG2^+^ ( *F* [3,7.122] = 2124.491, *p* < 0.001; Figure S3A,D,G), whereas none were SOX9^+^ (Figure S3A,E,H). The overwhelming OLIG2^+^ portion of HN^+^ Pop1 cells as compared to SOX9^+^ Pop1 cells (*H* (3) = 11.25, *p* = 0.010) remained intact in 8‐month‐old control animals (post hoc *p* = 0.030) and VWM animals alike, albeit non‐significant for the latter group (Figure 3H). At this age, a small portion of SOX9^+^HN^+^ cells was observed as well (Figure 3H). No differences between VWM and control mice were found in the cell fate of Pop1 transplants; the majority of transplanted Pop1 cells became OLIG2^+^. However, it should be noted that the few observed SOX9^+^HN^+^ cells were exclusively found in VWM mice.

The cell fate of Pop5 cells was also analyzed for expression of SOX9 or OLIG2 in vivo. HN^+^ Pop5 cells again more often expressed OLIG2 than SOX9 in 9‐month‐old transplanted mice (*F*[3, 19] = 14.14, *p* < 0.0005; Figure 3I) in both control animals (post hoc *p* = 0.001) and VWM animals (post hoc *p* = 0.019). At the same time, there was a significantly lower amount of OLIG2^+^HN^+^ cells in VWM animals compared with controls (post hoc *p* = 0.006, Figure 3I). No such difference between genotypes was seen in the amount of SOX9^+^HN^+^ cells. Thus, while oligodendrocytic cell fate was also the most prominent in HN^+^ Pop5 cells, the astrocyte cell fate upon transplantation was more prominent in HN^+^ Pop5 cells as compared to Pop1 cells. In addition, a larger portion of transplanted Pop5 cells were of a cell fate other than SOX9^+^ or OLIG2^+^ in VWM animals as compared to controls. These findings indicate that in vitro culturing approaches were reflected by in vivo cell fate and suggest some degree of cell‐intrinsic influence on cell fate.

The cell fate differences between Pop1 and Pop5 cells did not correlate to the prediction probability scores generated by the regression model (data not shown). That improvement of VWM phenotype was not a discriminatory factor in cell fate is in agreement with finding improved animals in both the Pop1 and Pop5 groups. Interestingly, mouse genotype did have an effect on the cell fate of Pop5 transplants, as indicated by a decreased OLIG2^+^ Pop5 cell fate in exclusively VWM mice. A similar effect was observed in the Pop1 cells, in which close to 100% of all transplanted Pop1 cells were OLIG2^+^, yet only in VWM animals, a small fraction of cells were SOX9^+^ instead. Consequently, cell fate of Pop1 and Pop5 cells was not linked to improvement status, but was instead affected by the microenvironment of the VWM brain.

### 3.5 Migration of HN ^+^ transplanted cells

**FIGURE 4:**
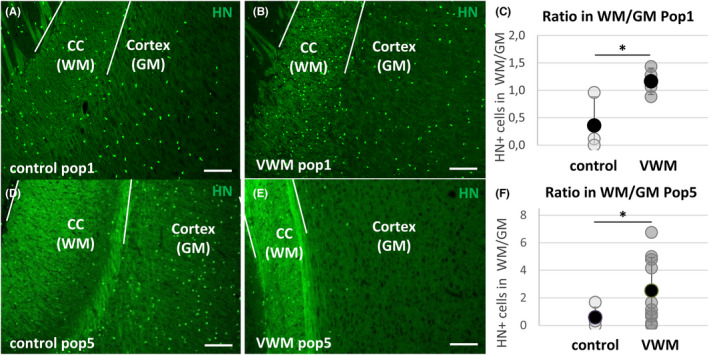
Differential density of transplanted cells in the white matter and gray matter. HN^+^ cell numbers were higher in the white matter (WM) than in the gray matter (GM) of VWM animals (A, D) than of control animals (B, E) at 8–9 months of age, irrespective whether animals were transplanted with Pop1 cells (C; VWM *n* = 4, controls *n* = 3) or Pop5 cells (F; VWM *n* = 10, controls *n* = 4). Independent t‐tests were used in C and D. *p < 0.05. Scale bars: 100 μm

To further study how the VWM microenvironment affected the transplanted cells, we analyzed the cell density in the white matter versus the gray matter in VWM animals and healthy controls (Figure 4). Specifically, the density of HN^+^ Pop1 cells was higher in VWM animals in the corpus callosum than in the cortex, and also when compared to either the cortex or the corpus callosum of heterozygous control animals (repeated‐measures ANOVA *F*[1, 18] = 5.306, *p* = 0.03; Figure S2B). Indeed, as compared to healthy controls (Figure 4A), the ratio of HN^+^ Pop1 cells in the white matter versus the gray matter was increased in VWM animals (independent *T* (5) = −2,77, *p* = 0.039; Figure 4B,C). Thus, while cells migrated to the entire brain, HN^+^ cells showed a preferential localization to the corpus callosum in VWM animals specifically. Although an ANOVA comparing both genotypes in both cell populations was not significant, the same effect was seen in transplanted Pop5 cells, for which the ratio of HN^+^ cells in the white over the gray matter was also higher in VWM animals as compared to controls (independent *T*[10.53] = −2.51, *p* = 0.030; Figure 4D–F). Thus, compared with healthy animals, the number of transplanted cells in VWM animals was higher in the white matter versus the gray matter, irrespective of whether Pop1 or Pop5 cells were transplanted. This suggests an effect of the VWM microenvironment on the spread of transplanted cells.

## 4 CONCLUSION

VWM is a severe neurodegenerative disease currently without effective treatments or curative options. The glial defects observed, particularly in the CNS of VWM patients and VWM mouse models, provide a relatively well‐defined target for cell replacement therapy. To follow up on a proof‐of‐concept study for glial cell replacement therapy,^11^ we tested human stem cell‐derived glial lineage cells appropriate for transplantation into the VWM mouse brain. Focusing on astrocytes in various stages of development, ranging from glial precursor cells to more mature astrocytes, the effects of cell transplants on VWM disease hallmarks were assessed. Transplanted human cells successfully migrated from the white matter injection sites throughout the brain, including the cortex, hippocampus, and brain stem, and showed astrocyte or oligodendrocyte lineage fates. When looking into various aspects of motor function and VWM disease markers, a number of VWM animals showed an improved VWM phenotype when transplanted with human ESC or iPSC‐derived glial populations (Pop1 or Pop5, respectively). This shows for the first time that human glial cell transplants can be used as a therapy for VWM. From the proof‐of‐principle study using mouse primary cells,^11^ we here provide an important translational step toward a treatment that may be used in VWM patients. Human stem cell technology allowed us to generate various cell populations and to select the best‐suited one, indicated by alleviation of disease severity upon transplantation. Our findings warrant further examination of graft‐brain microenvironment interactions, to benefit future cell replacement therapy approaches that might be human and brain region‐specific.

We hypothesized that cell populations primed for astrocytic cell fate would improve VWM phenotype severity, in line with the earlier proof‐of‐principle study using mouse glial precursor cells.^11^ While Pop1 and Pop5 both showed beneficial effects, preparative methods in vitro allowed Pop5 cells to develop into glial cell precursors with more astrocyte lineage cells as compared to Pop1 cells. Post‐grafting, this difference was reflected in the degree of oligodendrocytic, astrocytic, and alternative cell fates in vivo. Interestingly, the percentage of VWM animals that improved to control levels was not substantially larger in the Pop5‐treated group, despite its more astrocytic cell fate as compared to Pop1 cells upon transplantation. Thus, astrocytic cell fate in vivo does not seem to have a defining role in the success of cell replacement therapy when using human stem cell‐derived glial cells.

No discernable effect of maturation time in vitro was observed on the VWM phenotype, seeing that both Pop1 and Pop5 cells had beneficial effects when transplanted. However, Pop2 cells could not be traced back in vivo at 5 months post‐transplantation. These cells were matured for an extended period of time in astrocyte‐specific medium, and the poor graft survival could possibly result from lower plasticity of the cells at the time of transplantation. In comparison, the glial progenitor population grafts (pop1 and pop5) showed therapeutic benefits that may result from their increased plasticity, aiding their adaptation to the new microenvironment post‐transplantation. Our results thus indicate that immature cells show better survival in the host environment than do mature cells; the mechanism of graft‐induced phenotypic improvement is most likely primarily dictated by progenitor status in vitro. At the same time, Pop3 cells were matured for a shorter period in vitro than Pop1, Pop2, or Pop5, but did not result in phenotypic improvement. Among the glial progenitor populations treated with retinoic acid during in vitro preparation, a developmental window seemed to determine success. More in‐depth expression analysis of the retinoic acid‐induced cell populations might help to determine the identity of these successful populations as well as their developmental and molecular profile. Testing additional time points in vitro might identify more efficient graft populations. However, for therapeutic benefits in patients, species differences between the human and mouse microenvironment should be considered.

The transplanted cells interacted with the microenvironment in which they were transplanted. Between VWM and control animals, differences in the cell fate and spread of transplanted cells were observed. The degree of oligodendrocytic, astrocytic, or alternative cell fates did not only differ between transplanted cells, but also between VWM and control animals. In addition, a higher number of grafted cells in the white versus gray matter was observed in VWM animals as compared to healthy controls. This could suggest that affected VWM brain areas diminish the migratory capability of healthy transplanted cells. Alternatively, the diseased white matter may contain more chemo‐attractants that actively recruit transplanted cells. Similarly, it is difficult to determine to what extent the primarily oligodendrocytic cell fate signals a need of the VWM brain for myelin generation, or whether it reflects an intrinsic property of the transplanted cells. Potential alterations in endogenous oligodendrocyte precursor cell (OPC) differentiation following cell replacement therapy would be of interest. To study the maturation state of grafted HN^+^ vs. residential HN^−^ OPCs, we performed IHC‐based analysis of HN, OLIG2, and the mature oligodendrocytic marker myelin basic protein (data not shown). However, quantification proved unreliable and more in‐depth analysis of cell replacement therapy on local neural cell populations would need follow‐up in, for example, lineage‐tracking experiments. Together, our findings underscore a significant effect of the VWM brain microenvironment, in line with an earlier study suggesting that an as of yet unidentified secreted astrocytic factor is likely to contribute to VWM pathology.^6^ In agreement, our data suggest that the microenvironment affects how allogenic cells behave upon transplantation into the VWM brain.

Since the brain microenvironment of VWM mice was found to affect cell spread and cell fate post‐transplantation, future studies should also consider the human brain microenvironment in particular. Indeed, a role of the ECM has been shown in VWM with respect to the accumulation of hyaluronic acid (HA) in both patient white matter^27^ and in VWM mice.^6^ In vitro studies showed that hyaluronidase (HAse) treatment restored primary mouse OPC maturation defects when cultured in human iPSC‐derived VWM astrocyte conditioned medium (ACM).^28^ Interestingly, HAse treatment of VWM mouse ACM did not improve OPC maturation.^6^ This suggests that additional astrocytic factors might be involved and also indicates differences between the human and mouse VWM microenvironment. Age might additionally be an important factor in microenvironmental changes, both during normal development^29^, ^30^ and in a progressive neurodegenerative disease such as VWM.^31^, ^32^, ^33^ It would be interesting to see whether the cell populations tested here can be of benefit when transplanted at a later age, a scenario relevant to many VWM patients. Changes in the microenvironment over time could call for adaptations to the transplanted cell population. So, future research into the brain microenvironment specific to the VWM patient, and its effects on transplanted cells, should be considered.

In conclusion, we show that human stem cell‐derived glial cell transplants can have regenerative effects for VWM. Of various cell populations tested, transplantation of mixed glial cell populations showed therapeutic benefits. The VWM genotype affected cell fate and the spread of these cells, indicating an interaction effect with the microenvironment. Taking this into consideration, finding the appropriate conditions for cell replacement therapy in VWM patients might fall in the realm of personalized medicine, requiring a Goldilocks graft that is “just right.”

## AUTHOR CONTRIBUTIONS

A.E.J.H., P.S.L., N.B.B., S.D., and T.B. conducted experiments and gathered data. A.E.J.H., P.S.L., N.B.B., S.D., and T.B. analyzed the data. A.E.J.H. wrote the manuscript. V.M.H. and M.v.d.K. contributed to study design, provided supervision, materials and resources, and revised the manuscript. A.E.J, P.S.L., S.D., and N.B.B. revised the manuscript.

## CONFLICT OF INTEREST

The authors have no financial support or relationships that pose conflicts of interest to disclose.

## Supporting information


Figure S1

Figure S2

Figure S3

Appendix S1

Appendix S2

## Data Availability

The data that support the findings of this study are available from the corresponding author upon reasonable request.
