# Supplementary figures and images for: Therapeutic potential of human stem cell transplantations for Vanishing White Matter: A quest for the Goldilocks graft
Source: CNS Neurosci Ther. 2022 Jul 1;28(9):1315–25. doi: 10.1111/cns.13872 (PMC9344080; doi:10.1111/cns.13872)

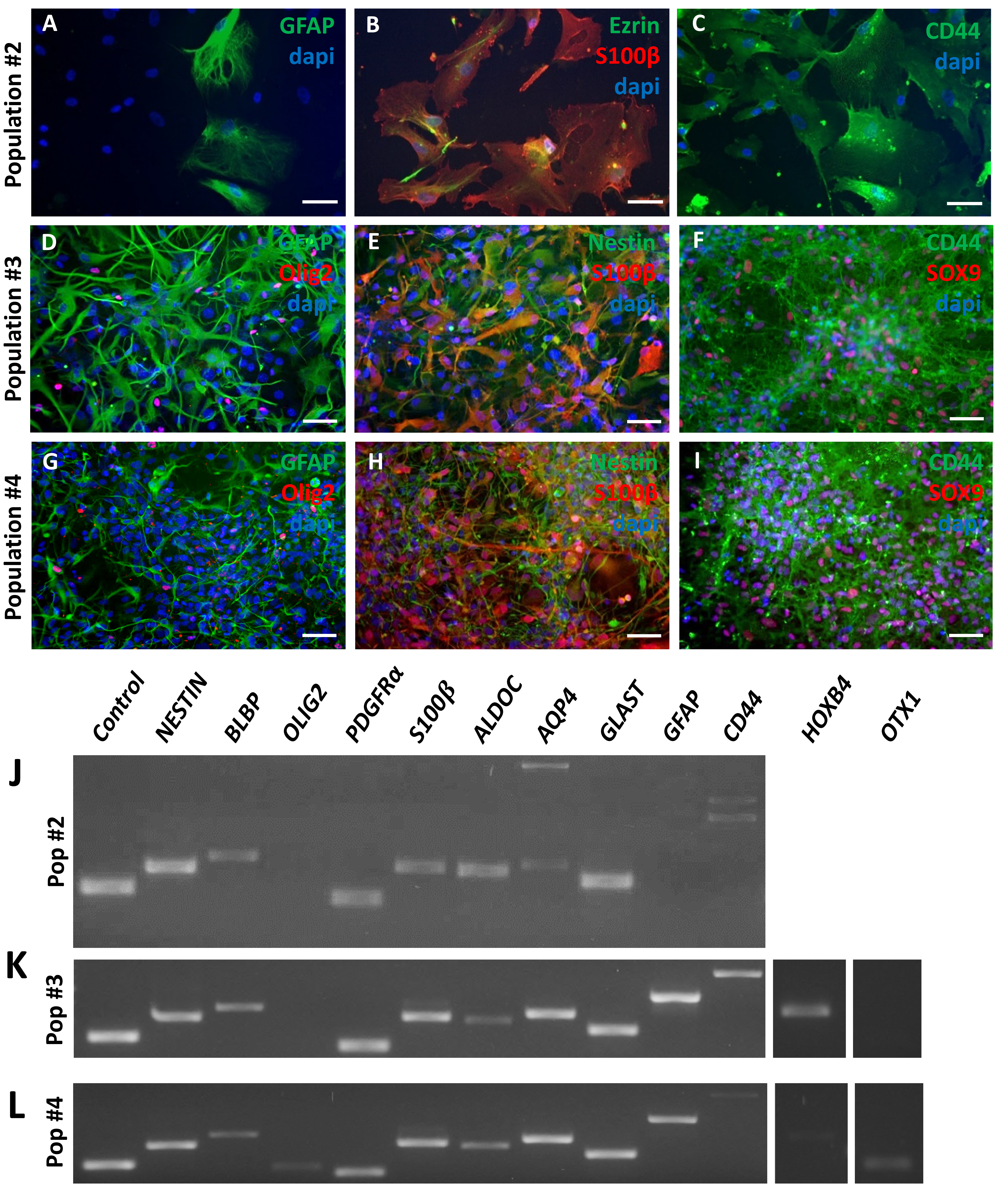

Supplement: Supplementary file 1 — Figure S1 [file CNS-28-1315-s005.tif]

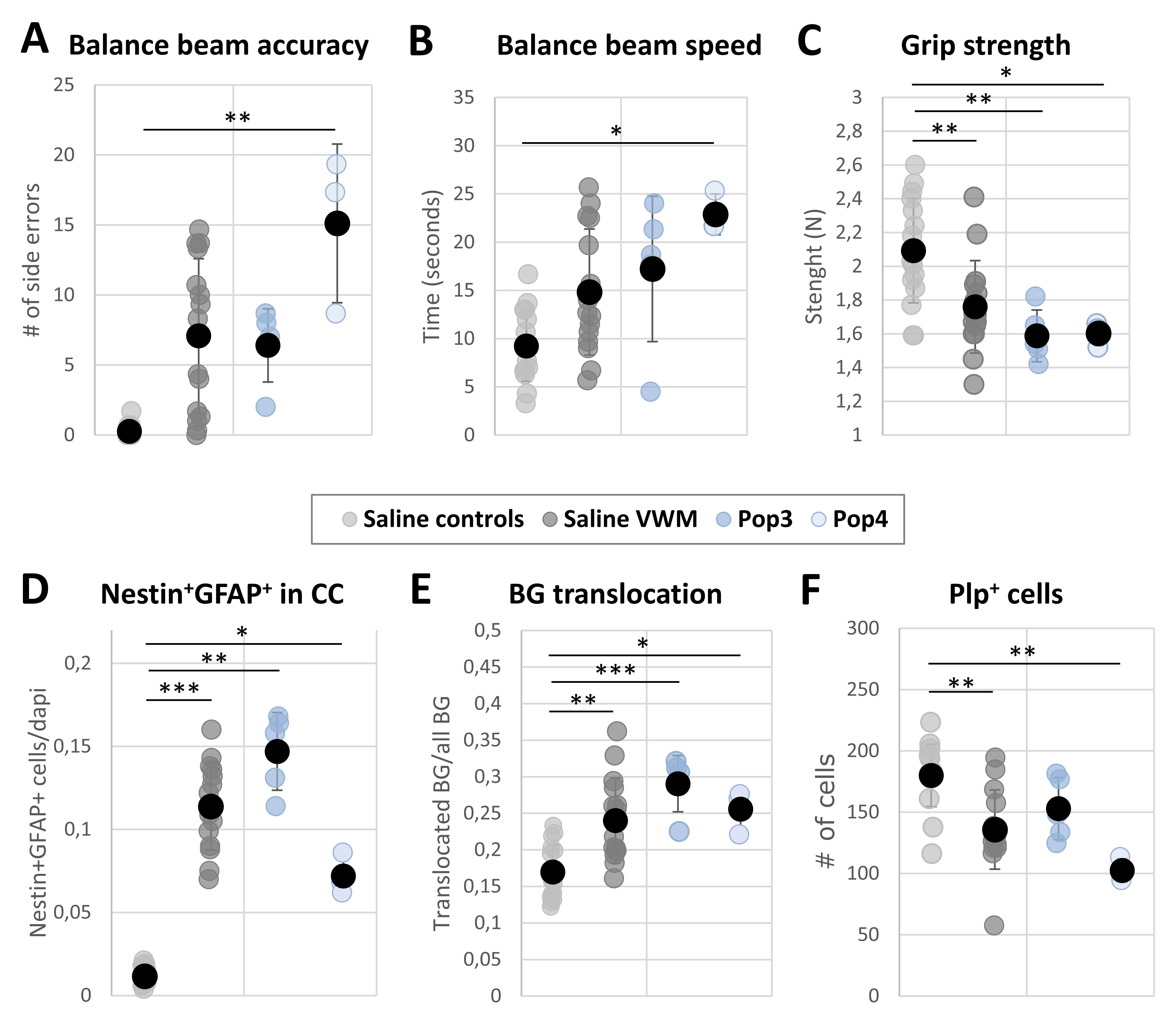

Supplement: Supplementary file 2 — Figure S2 [file CNS-28-1315-s004.tif]

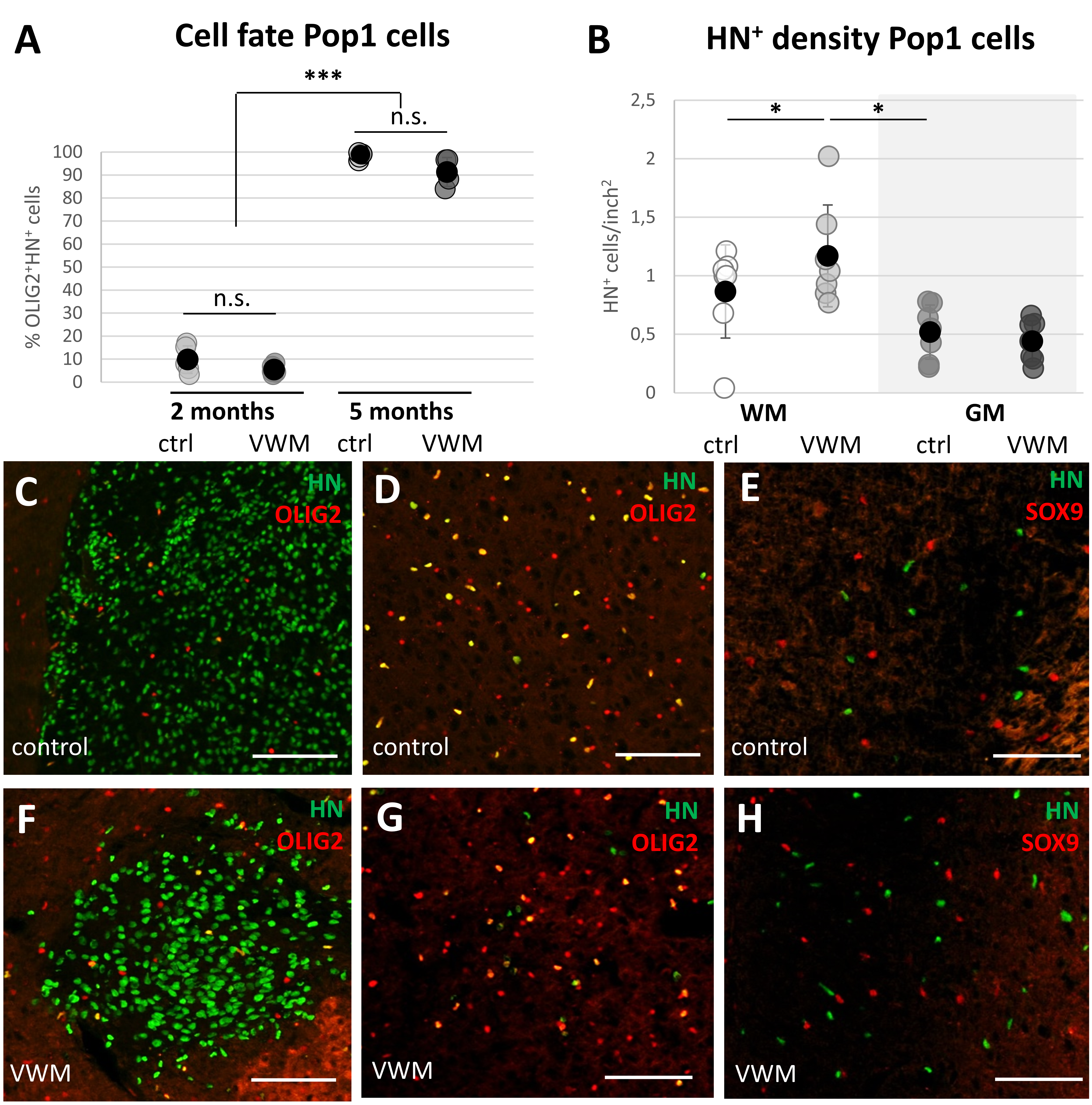

Supplement: Supplementary file 3 — Figure S3 [file CNS-28-1315-s001.tif]
