# Supplementary material for: Therapeutic potential of human stem cell transplantations for Vanishing White Matter: A quest for the Goldilocks graft
Source: CNS Neurosci Ther. 2022 Jul 1;28(9):1315–25. doi: 10.1111/cns.13872 (PMC9344080; doi:10.1111/cns.13872)
Supplement: Supplementary file 4 — Appendix S1 [file CNS-28-1315-s002.docx]

**Therapeutic potential of human stem cell transplantations for Vanishing White Matter: a quest for the Goldilocks graft**Hillen, A.E.J.; Leferink, P.S.; Breeuwsma, N.B.; Dooves, S.; Bergaglio, T.; Van der Knaap, M.S.; Heine, V.M.

**Supplementary Information**

*Glial progenitor differentiation*

Glial progenitor cells were generated from human embryonic stem cells (hESCs) using adapted versions of published protocols ^1,2^. After detaching and fragmenting hESC colonies with 0.5mM EDTA in PBS, cells were transferred 2:1 to an anti-adhesive (AA) poly-2 hydroxyethyl methacrylate (Sigma)-coated plate. Here, cells were allowed to form embryoid bodies (EBs) in N2B27 medium (1:1 mixture of N-2 medium (DMEM/F-12 GlutaMAX, 1× N-2, 5μg ml−1 insulin, 1mM L-glutamine, 100μM non-essential amino acids, 100μM 2-mercaptoethanol, 50U mL−1 penicillin and 50mg mL−1 streptomycin) and B-27 medium (Neurobasal, 1× B-27, 200mM L-glutamine, 50U mL−1 penicillin and 50mg mL−1 streptomycin), supplemented with fibroblast growth factor 2 (FGF2; 4ng/mL), epidermal growth factor (EGF; 20ng/mL) and Rock inhibitor (RI; 10μM). Half of the medium was refreshed every other day. RI was replaced with Retinoic Acid (RA; 10μM) after two days of EB formation. EBs were plated on GelTrex-coated plates at day 10, and were incubated with N2B27 medium supplemented with EGF (20ng/mL). Eight days after plating, cells were passaged using Accutase. After this first passage during the adhesive stage, cells were cultured in N2B27 medium composed of the same reagents but with B27-vitamin A instead of regular B27. This N2B27-VitA medium was supplemented with EGF (20ng/mL).
 The cells of population **#1** were passaged 5 more times for expansion, and frozen at day 53 in Dulbecco’s modified Eagle’s medium (DMEM)/F12 with 20% Knock-out serum replacement and 10% DMSO. Two days before injection, the cells were thawed, and cultured in a similar way. Cells of population **#5** were differentiated using the same protocol, but were maintained in N2B27-VitA medium supplemented with EGF (20ng/mL) and T3 (40ng/mL) until transplantation at 60-105 days.

*Astrocyte differentiations*

In addition to the glial progenitor populations, various astrocyte differentiations (**Table S1**) were performed as described previously ^2^. Populations varied in the induction method used during the first 18 days of the protocol; in cell origin; and in medium post-differentiation. After the 18 day induction period, a glial differentiation protocol ^2^ with slight adaptations was used to generate astrocytes. Briefly, cells were grown on plates coated with GelTrex in N2B27-VitA medium supplemented with T3 (40ng/mL) and EGF (20ng/mL). Medium was fully refreshed every other day. Cells were passaged with Accutase as required. After about 4 passages, about 20 days (37 days in culture in total), cells were grown in N2B27-vitA medium supplemented with T3 (40ng/mL), EGF (5ng/mL), FGF2 (5ng/mL), Noggin (50ng/mL, Peprotech), vitamin C (50μg/mL, Sigma), and laminin (1μg/mL, Sigma). EGF and FGF2 were omitted from the medium after 5 days, at day 42 in the protocol. A brief outline of the 18 day induction used per cell population is described below.

For populations #2-4, cells were cultured in the following manner:
**#2**: similar protocol as population #1. Briefly, induced pluripotent stem cells (iPSCs) were fragmented and allowed to form EBs. EB formation was done in N2B27 medium supplemented with RI, EGF, and FGF2. RI was switched with RA after 2 days. At day 10, EBs were transferred to adhesive plates and passaged as needed using Accutase. Cells were expanded in N2B27-VitA medium supplemented with T3 and EGF, and cultured in N2B27-VitA medium supplemented with T3, EGF, FGF2, Noggin, vitamin C, and laminin for 5 days before switching to commercial astrocyte medium at day 42 in the protocol. Cells were maintained in this medium until day 112, at which time they were transplanted.
**#3**: generated from an iPSC line from a healthy control and cultured like #1. Cells were cultured in adhesive stage in N2B27 + EGF (20ng/mL) as described for #5, until day 42. At this time point, cells were prepared for transplantation.
**#4**: generated from hESC line H01. Colonies were fragmented and transferred to AA plates, as with population #1. N2B27 medium was supplemented with RI and FGF, not using EGF. After daily refreshing of two-thirds of the medium, RI was omitted at day 3. EBs were plated and passaged as with #1-3. Cells were kept in differentiation medium (as population #2-3) until day 42 of the protocol when the cells were transplanted.

| Table S1. Overview of cell populations generated for transplantation. | | | | |
| --- | --- | --- | --- | --- |
| Population | Induction | Culturing protocol | Source | N mutants* |
| #1 | RA | Matured in final differentiation medium until day 53 | ESC H01 | 4 (5) |
| #2 | RA | Matured in commercial astrocyte medium until day 112 | ESC H01 | 0 (7)** |
| #3 | RA | Matured in differentiation medium until day 42 | iPSC | 5 (5) |
| #4 | FGF2 | Matured in differentiation medium from day 42 onwards | ESC H01 | 3 (3) |
| #5 | RA | Matured in final differentiation medium until day 60-105 | iPSC | 9 (12) |

*: number of animals that were included in the regression analysis; the total number of injected animals of this age group is shown in brackets.
**: no HN^+^ cells were found in animals transplanted with this population and animals were therefore excluded from further analysis.


*Immunohistochemistry*

Cell cultures were characterized using immunocytochemistry (ICC). Cells cultured on cover slips were washed with PBS and fixated in 4% PFA for 15’. Cover slips were then washed in PBS for 3x 10’ with agitation, and incubated in blocking buffer (5% normal goat serum, 0.1% Triton-X, and 0.3% bovine serum albumin in PBS) for 1 hour at room temperature (RT). Primary antibodies (**Table S2**) were diluted in blocking buffer and incubated at room temperature for 1 hour prior to overnight incubation at 4°C. The next day, cells were washed in PBS for 3x 10’, and incubated for 1h at RT with secondary antibodies diluted in blocking buffer. Cells were washed once more for 3x 10’, incubated with dapi (Sigma, 25ng/mL) for 1 minute, and washed one final time before embedding the cover slips in Fluoromount-G (Southern Biotech) onto a microcopy slide.
 Sectioned brain tissue of 12µm thick was used for immunohistochemistry (IHC). Sections were washed in PBS 6x 5’ with agitation before receiving heat-mediated antigen retrieval in 0.01M citrate buffer (0.1M citric acid and 0.1M sodium citrate 1:10 in MQ). Slides were put in hot (~90°C) citrate buffer and kept at ~90°C for 10’ before cooling down. After 40 minutes, slides were washed in PBS once and incubated in blocking buffer for 1h at RT. Blocking buffer was then removed and primary antibodies diluted in blocking buffer were added to slides for 1h at RT before incubation overnight at 4°C. The next day, slides were washed 6x 5’ in PBS. Secondary antibodies were diluted in blocking buffer and slides were incubated for 2h at RT. Slides were again washed 6x 5’ in PBS before incubating with dapi (25ng/mL) for 2-5 minutes. After a final PBS wash, Fluoromount-G was added and slides were covered with a cover glass.

*RT-PCR*

To characterize cells *in vitro,* RT-PCR was performed as described previously.^2^ Briefly, RNA was isolated using TRIzol reagent based on the manufacturer’s instructions (Invitrogen). cDNA was generated for 1µg of RNA using Superscript III RT (200U/µL, Invitrogen) according to manufacturer’s instructions. 1µL of cDNA was used together with Phire III DNA polymerase and appropriate buffer components to generate PCR products using primers listed in **Table S3**. The PCR program consisted of 1 minute at 98°C, then 30 cycles of 15 seconds at 95°C, 15 seconds at 60°C, and 15 seconds at 72°C, finished by 30 seconds at 75°C. Samples were then cooled down to 4°C. 2% agarose (Roche) was dissolved in TBE and 1µL/mL ethidium bromide (Sigma). The gels were run at constant 75V for about 60 minutes.

*In situ hybridization (ISH)*

*In situ* hybridization for *Plp* mRNA was performed as described by Dooves *et al*.^3^ Briefly, brain sections were treated with proteinase K before a dig-labeled probe was incubated in hybridization buffer overnight. The Plp probe was targeted with anti-digoxygenin-AP (1:2000, Roche). Staining using BM Purple (Roche) was allowed to develop overnight. Tissue was counterstained with 0.5% methyl green.


Primary antibodies used for IHC and ICC are described in Table S2. Primers used for RT-PCRs for *in vitro* characterization are listed in Table S3. Descriptive statistics per assay per group are listed in Table S4. Details of the regression model are described in Table S5.

| **Table S2.** Antibodies used for ICC and IHC. | | | | |
| --- | --- | --- | --- | --- |
| **Antigen** | **Company** | **Host** | **Dilution** | **Application** |
| Human Nucleus | Millipore | Mouse | 1:500 | IHC |
| OLIG2 | Millipore | Rabbit | 1:1000 | IHC, ICC |
| SOX9 | Cell Signalling | Rabbit | 1:500 | IHC, ICC |
| GFAP | DAKO | Rabbit | 1:1000 | IHC, ICC |
| NESTIN | BD Bioscience | Rabbit | 1:500 | IHC, ICC |
| CD44 | Hybridomabank | Mouse | 1:100 | ICC |
| S100β | ProteinTech | Rabbit | 1:1000 | ICC |

| **Table S3.** Primer sequences used for RT-PCR. | | |
| --- | --- | --- |
| **Gene** | **Forward primer sequence** | **Reverse primer sequence** |
| *EIF4G2 (housekeeping)* | ATTCTTCGTTGTCAAGCCGCCAAAGTGGAG | AGTTGTTTGCTGCGGAGTTGTCATCTCGTC |
| *NESTIN* | CAGGAGAAACAGGGCCTACA | TAAGAAAGGCTGGCACAGGT |
| *BLBP* | GGATTGGGAGGAACTCGACC | CCCACGCCTAGAGCCTTCAT |
| *S100β* | GGTGAGACAAGGAAGAGGATGT | ACAGGAAAGGTTTGGCTGCT |
| *ALDOC* | CCATGCCTGTCCCATCAAGT | TGCAAGCCCATTCACCTCAG |
| *AQP4* | AAGGCGGTGGGGTAAGTGTG | CACTGGGCTGCGATGTAGAA |
| *GLAST* | ACATGAAGGAACAGGGGCAG | ACCCAAGGGTTTTTCCGTGT |
| *GFAP* | GCAGATTCGAGAAACCAGCC | GAGGGCGATGTAGTAGGTGC |
| *hCD44* | TTACAGCCTCAGCAGAGCAC | AGGTGGAGCTGAAGCATTGA |
| *hSOX2* | CATCACCCACAGCAAATGAC | TTTTTCGTCGCTTGGAGACT |
| *hPDGFR-α* | GAAGCTGTCAACCTGCATGA | CTTCCTTAGCACGGATCAGC |
| *hOLIG2* | TCGCATCCAGATTTTCGGGT | AAAAGGTCATCGGGCTCTGG |
| *hMBP* | CAGGGAAAGGGGAGAGGACT | TGGGTGATCCAGAGCGACTA |
| *hHOXB4* | GTGAGCACGGTAAACCCCAAT | CGAGCGGATCTTGGTGTTG |
| *hOTX1* | CACTAACTGGCGTGTTTCTGC | GGCGTGGAGCAAAATCG |

**Table S4.** Descriptive statistics of assays per group.

| **Assay** | **Group*** | **Mean + standard deviation** | **95% interval** |
| --- | --- | --- | --- |
| Balance beam speed | **Pop3 Pop4** | M = 17.23 ± 7.53 M = 22.89 ± 2.11 | 7.88 – 26.59 17.64 – 28.14 |
| Balance beam accuracy | **Pop3 Pop4** | M = 6.4 ± 2.62 M = 15.11 ± 5.67 | 3.15 – 9.65 1.03 – 29.19 |
| Grip strength | **Pop3 Pop4** | M = 1.588 ± 0.154 M = 1.603 ± 0.073 | 1.397 – 1.779 1.420 – 1.786 |
| Nestin^+^ astrocytes in the corpus callosum | **Pop3 Pop4** | M = 0.147 ± 0.023 M= 0.072 ± 0.012 | 0.118 – 0.176 0.041 – 0.103 |
| Bergmann glia translocation | **Pop3 Pop4** | M= 0.2904 ± 0.039 M= 0.2557 ± 0.030 | 0.243 – 0.339 0.181 – 0.331 |
| *Plp*^+^ expression in the white matter | **Pop3 Pop4** | M= 152.93 ± 25.35 M= 102.50 ± 9.69 | 121.45 – 184.41 78.43 – 126.57 |
| Cell fate Olig2^+^ | **Pop1** 2 months control VWM 5 months control VWM 8 months control VWM **Pop5** 9 months control VWM | M = 9.80 ± 5.95 M = 5.43 ± 2.02  M = 98.31 ± 1.43 M = 91.31 ± 6.34  M = 98.67 ± 2.31 M = 96.00 ± 0.82   M = 78.00 ± 9.90 M = 50.11 ± 14.29 | 2.41 – 17.18 2.92 - 7.94   96.54 – 100.08 81.23 – 101.41  92.93 – 104.40 94.70 – 97.30   62.25 – 93.75 39.13 – 61.09 |
| Cell fate Sox9^+^ | **Pop1** 8 months control VWM **Pop5** 9 months control VWM | M = 0.00 ± 0.00 M = 7.50 ± 0.96   M = 32.25 ± 6.70 M = 29.17 ± 5.70 | 0.000 – 0.000 -0.80 – 2.27   21.59 – 42.91 23.18 – 35.16 |
| Cell localization (white matter/grey matter ratio) | **Pop1** control VWM **Pop5** control VWM | M = 0.358 ± 0.524 M = 1.165 ± 0.241  M = 0.585 ± 0.744 M = 2.517 ± 2.420 | -0.944 – 1.660 0.782 – 1.55  -0.599 – 1.769 0.786 – 4.25 |
| Cell density per inch^2^ | **Pop1** WM control VWM GM control VWM | M = 0.866 ± 0.398 M = 1.170 ± 0.435  M = 0.519 ± 0.232 M = 0.440 ± 0.175 | 0.498 – 1.234 0.768 – 1.572  0.304 – 0.733 0.279 – 0.601 |

*If already included in Table 1, data of saline-treated healthy controls, saline-treated VWM animals, Pop1-treated VWM animals, and Pop5-treated VWM animals are omitted. Pop3 and pop4 groups only include VWM animals.

**Table S5**. Overview of the prediction variables included in the logistic regression model and the classification scores of training and test groups.

| *The logistic regression model compiles a binary (VWM or control) score based on:* | | |
| --- | --- | --- |
| Motor function assays | Grip strength of the paws | |
|  | Balance beam speed | |
|  | Balance beam accuracy | |
| VWM brain pathology | Translocated Bergmann glia | |
|  | Nestin^+^ astrocytes in the corpus callosum | |
|  | PLP^+^ cells in the corpus callosum and cerebellar white matter | |
| *Logistic regression scores:* | | |
| Saline-treated VWM animals (training group) | used to train the model on VWM classification | Score = 0.00000 |
| Saline-treated control animals (training group) | used to train the model on control classification | Score = 1.00000 |
| Cell-treated VWM animals (test group) | classified by trained model as VWM or control based on cut-off threshold of score | Score ≥ 0.75000: performance considered similar to controls; improved |

**References for Supplemental Information**

1. Izrael M, Zhang P, Kaufman R, Shinder V, Ella R, Amit M, et al. Human oligodendrocytes derived from embryonic stem cells: Effect of noggin on phenotypic differentiation in vitro and on myelination in vivo. *Mol Cell Neurosci.* 2007;34(3):310-323.

2. Nadadhur AG, Leferink PS, Holmes D, Hinz L, Cornelissen-Steijger P, Gasparotto L, et al. Patterning factors during neural progenitor induction determine regional identity and differentiation potential in vitro. *Stem Cell Res.* 2018;32:25-34.

3. Dooves S, Leferink PS, Krabbenborg S, Breeuwsma N, Bots S, Hillen AEJ, et al. Cell Replacement Therapy Improves Pathological Hallmarks in a Mouse Model of Leukodystrophy Vanishing White Matter. *Stem Cell Reports.* 2019;12(3):441-450.
