# Supplementary material for: Therapeutic potential of human stem cell transplantations for Vanishing White Matter: A quest for the Goldilocks graft
Source: CNS Neurosci Ther. 2022 Jul 1;28(9):1315–25. doi: 10.1111/cns.13872 (PMC9344080; doi:10.1111/cns.13872)

Full unedited gel for Figure 3

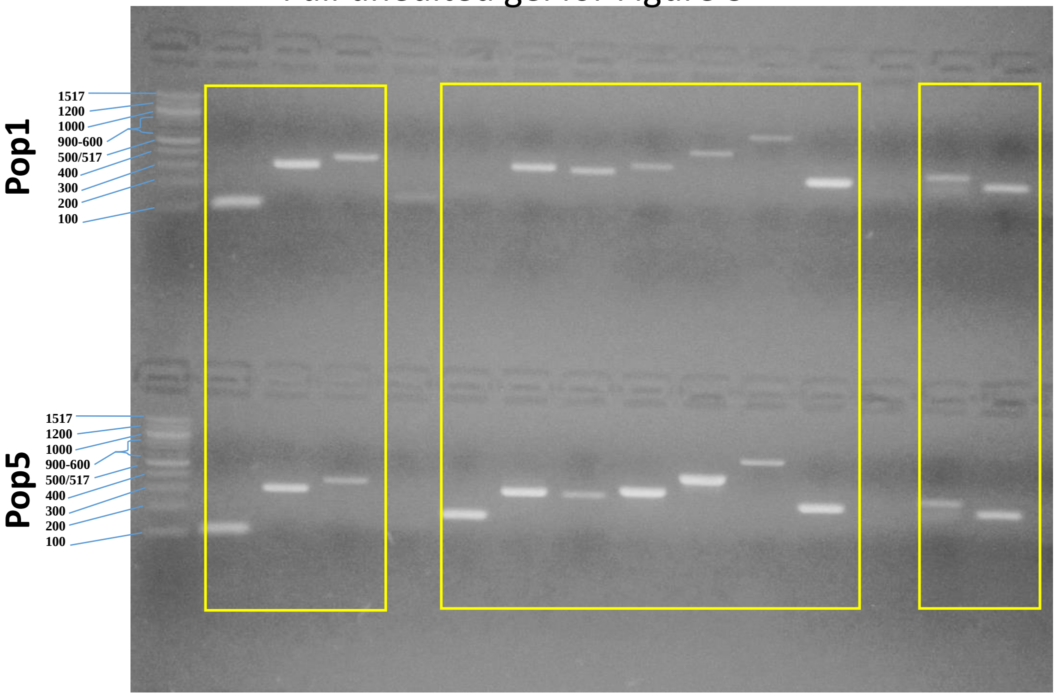

New England Biolabs ladder N3132#:

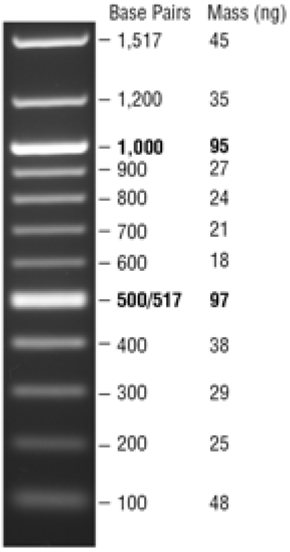

Full unedited gels for Supplemental figure S1

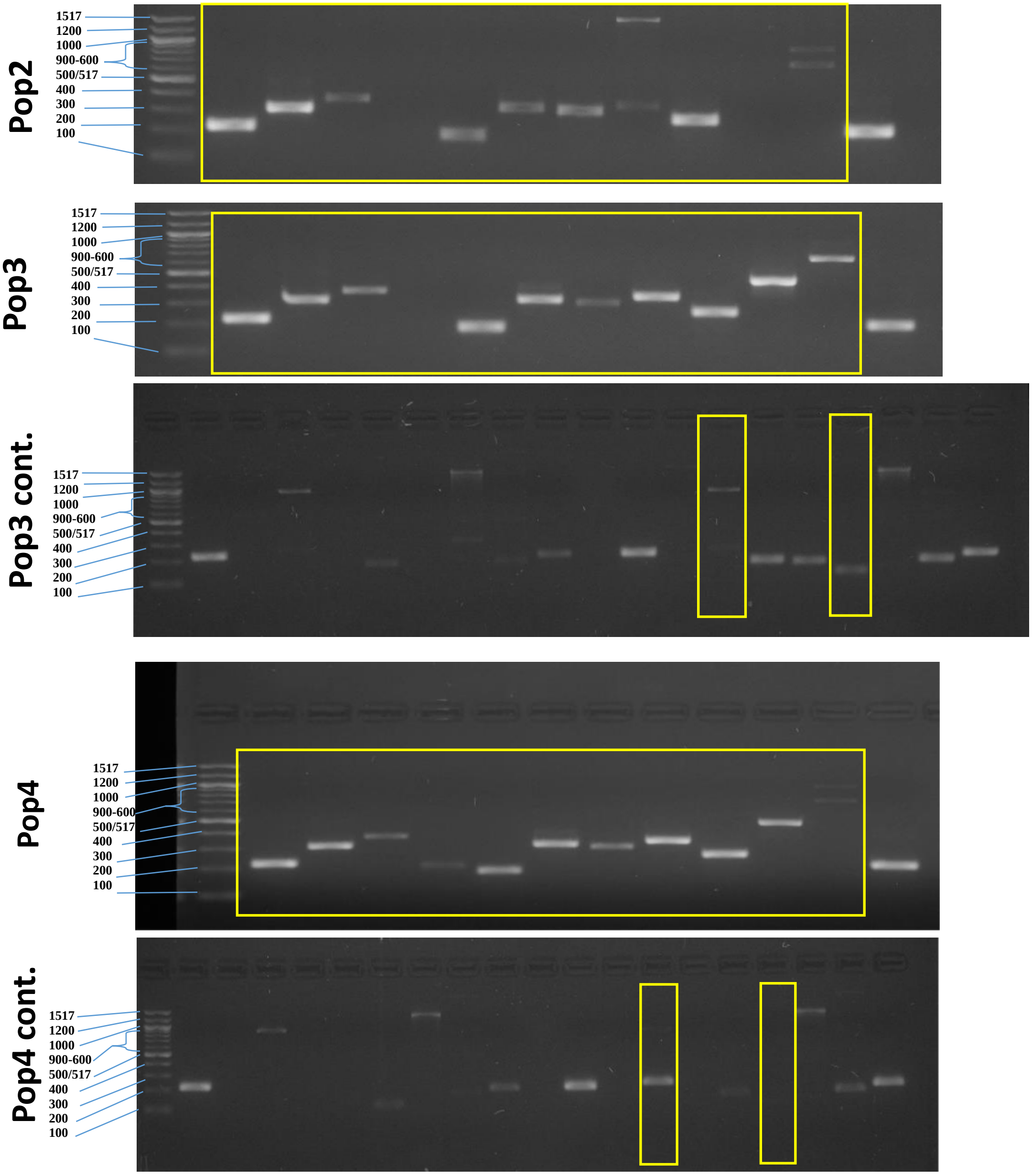

Supplement: Supplementary file 5 — Appendix S2 [file CNS-28-1315-s003.pdf]
